# Flight flexibility in strategic traffic planning: visualisation and mitigation use case

**DOI:** 10.1007/s13272-021-00541-7

**Published:** 2021-08-30

**Authors:** Tatjana Bolić, Lorenzo Castelli, Giovanni Scaini, Giuseppe Frau, Stefano Guidi

**Affiliations:** 1 Università degli Studi di Trieste, Trieste, Italy; 2 University of Westminster, London, UK; 3 Deep Blue, Rome, Italy; 4 University of Siena, Siena, Italy

**Keywords:** Strategic traffic planning, Time windows, European air traffic management, Data visualization, Mitigation action, Assessment

## Abstract

The concept of strategic traffic planning that takes into account changing airspace configurations, their capacity, and allows the quantification of flight flexibility is presented in this paper: the visualization of the results and an example of possible use. The concept is implemented through two deterministic optimization models. Here, we focus on the output of the models, which identifies the departure times, trajectories, flight flexibility and the list of saturated sector-hours throughout the day, based on the configurations used during the day. In order to make the output understandable to various stakeholders, we use a visualization tool and a set of performance indicators. The information on the saturated sectors, and their impact on flexibility (criticality index) is taken as an input in the example of mitigation action application by Air Navigation Service Providers, aimed at improving the situation. A mitigation strategy of increasing capacity of saturated airspace is implemented, and results show that the improvements in flexibility can be achieved.

## Introduction

At the time of the writing, the air traffic in Europe is slowly picking up from 90% decrease due to COVID-19 crisis. Past few weeks are registering about 40% of traffic when compared to 2019.^1^ In the past decades the air transport demand often rebounded after external shocks, like 9/11, 2008 financial crisis, or 2010 volcanic eruptions in Europe [1]. Thus, we might hope that at some point in the future the air traffic will return to 2018 or 2019 levels. In the meantime, we could prepare for such capacity crunch, improving the collaborative decision making capabilities of European air traffic management (ATM) network. The previous period of decrease in flights (between 2008 and 2013) was followed by a steady increase, on average 3% annually. In June 2019 the record number of daily flights was recorded in the European airspace (on average about 36,000 flights per day), which was accompanied by the increase of delays—only between 2017 and 2018 there was a 61% increase in delay peaks [2]. En-route air traffic flow management (ATFM) accounted for the 74% of total ATFM delay, main contributing causes being en-route air traffic control (ATC) capacity (28%), weather (19%) and en-route ATC staffing (17%). The ATC capacity and ATC staffing^2^ delays are usually caused by misalignment of information exchange between the airspace users (on the exact flight demand) and the air traffic management (ATM) capacity.

In the current setup, high level of flight planning flexibility exists as the flight plans have to be submitted 3 h before departure at the latest [3]. From the conversations with the scheduled airlines’ representatives, we understand that flight plans rarely get submitted more than 12 h before the departure, which offers a flexibility to create flight plans that take into account various not easily foreseen factors, like the weather forecasts or specific aircraft availability. On the contrary, the Air Navigation Service Providers (ANSPs) plan the capacity provision (e.g. staffing levels) about a year before, updating it over time. This flexibility comes at the cost as it makes the traffic demand on ATM system less predictable (the precise demand is known only on the day of operations), and creates capacity-demand imbalances that result in the ATFM measures which impose delays and consequently costs, estimated to be more than 1.9B€ in 2018 [4]. Furthermore, the airlines do not need to take into account the capacity of the network elements, nor do they have that information available during the creation and subsequent submission of a flight plan.

Most of the literature on airspace congestion addresses tactical problems, ATFM problem being studied the most. The ATFM problem aims to define ground and airborne holding, and rerouting actions in order to solve capacity-demand imbalance on the day of operations. Odoni [5] was the first to formalise the model, and a number of studies built on this work (see for example Bertsimas and Stock Paterson [6, 7], Andreatta and Odoni [8], Lulli and Odoni [9] and Bertsimas et al. [10]). Of course, a variety of tools for addressing ATFM problems are in use in different parts of the world. The ample body of research and development focuses on improving their efficiency (as an example: Brasil et al. [11], or Ruiz et al. [12]).

Here, we focus on the strategic planning phase^3^, significantly earlier than the tactical applications. Studies dealing with the strategic capacity-demand imbalances can be divided in those introducing pricing mechanisms (see [8], Jovanović at al. [13], Bolić at al. [14]), or managing air traffic capacity under demand and capacity provision uncertainty (see Starita at al. [15]).

Earlier information exchange (from 6 months to few days before the operations) on the flight trajectories and the available airspace capacity could help close this information gap and reduce the amount of ATFM delays, especially taking into account that the European ATM Master Plan [16] envisions such collaborative exchange through the Network Operations Portal. Bolić et al. [17] show that earlier information exchange can reduce delays. They developed an integer programming model for strategic flight planning, which uses past and early-shared trajectory information to distribute the traffic in a way that respects the declared nominal capacities of airports and sectors on the entire European network. Consequent decrease of demand capacity imbalances at the strategic planning level leads to a reduction of the number of ATFM interventions (mainly ATC capacity reason) on the day of operations. The results of this model, coupled with results of a second, time windows model, are used in this work. The notion of time windows is not new (see Berechet et al. [18], Han et al. [19], Margellos and Lygeros [20] or Rodriguez at al. [21]), but it addresses the execution, or tactical planning phases of a flight, attempting to assess the precision of the trajectory execution.

The objective of this research is twofold. On the one hand, we propose a visualisation tool for representing results of complex models, aimed at different stakeholders: airlines, ANSPs, NM, airports. On the other hand we investigate how the early sharing of information on traffic demand (i.e. trajectories) and planned ATC capacity would enable the early, collaborative capacity and demand planning, and what type of benefits could be achieved. In other words, novel contribution consists of addressing the strategic traffic planning (as opposed to the tactical one), flight flexibility quantification, and presenting the results of these planning models in a simple way, intended for use by different aviation stakeholders. For the purposes of this paper, the flight flexibility is defined as time intervals (i.e. time windows) around the planned departure, arrival, or times of entry into sectors along the route; as long as the trajectory is operated within this window, it will not cause disturbances to other flights in the system (e.g. delay). As the first step, we present the visualisation of the strategic traffic planning models. In the second step, as implementing the strategic planning would represent the change in the current way of working, we provide an assessment of such a concept. The assessment consists of comparison between the baseline (i.e. current operations), solution and mitigation scenarios. Solution scenario implies the application of strategic planning models on the initial input data, while mitigation scenario presented here consists of adjustment of capacity provision.

Section 2 describes the strategic traffic planning and flexibility concept, gives an example of the time window application, describes the data instance and run times of the described models; Section 3 describes the indicator devised for easier understanding of results and gives an overview of the information that can be obtained from visualization tool. Section 4 describes the example of use of the concept and the visualization tool, followed by Sect. 5 that shows the assessment of the chosen mitigation action, and Sect. 6 concludes the work.

## Strategic traffic planning and flexibility concept

In this work, we describe the visualization and a possible use of results coming from the strategic traffic planning, and the flight flexibility models. The concept aims at enhancing predictability of traffic planning through earlier sharing of information, and quantifying the flexibility flights would have when doing so. To do so we developed two models (details of which can be found in the associated references):

- Strategic air traffic assignment (SATA)^4^ [17],
- Time Windows (TW) model [22]

In the first step, SATA assigns the scheduled/planned departure time and trajectory (in the form of subsequent sector entry times) for each flight, minimising overall (i.e. system optimum) flights’ strategic operational costs, subject to the capacity of network elements (i.e. airports and sectors). The cancellations are not allowed and speed control is not taken into account, as it would make little sense in the strategic phase. The maximum allowed difference between the assigned (by the model) and originally requested times for departure/arrival times is bounded, and airspace configuration^5^ changes (with associated capacities) throughout the day are taken into account. Since speed control is not taken into account, departure time and route information enable determining the time of entry into sectors along the trajectory and the arrival time for each flight. In this step, the traffic is distributed across the network respecting the declared capacities, and as such, providing predictability for ANSPs. However, the 4D trajectories assigned by the model could be interpreted to mean that all the flights must adhere exactly to the specified timings along the trajectory, which would drastically reduce the airlines’ flexibility.

**Fig. 1:**
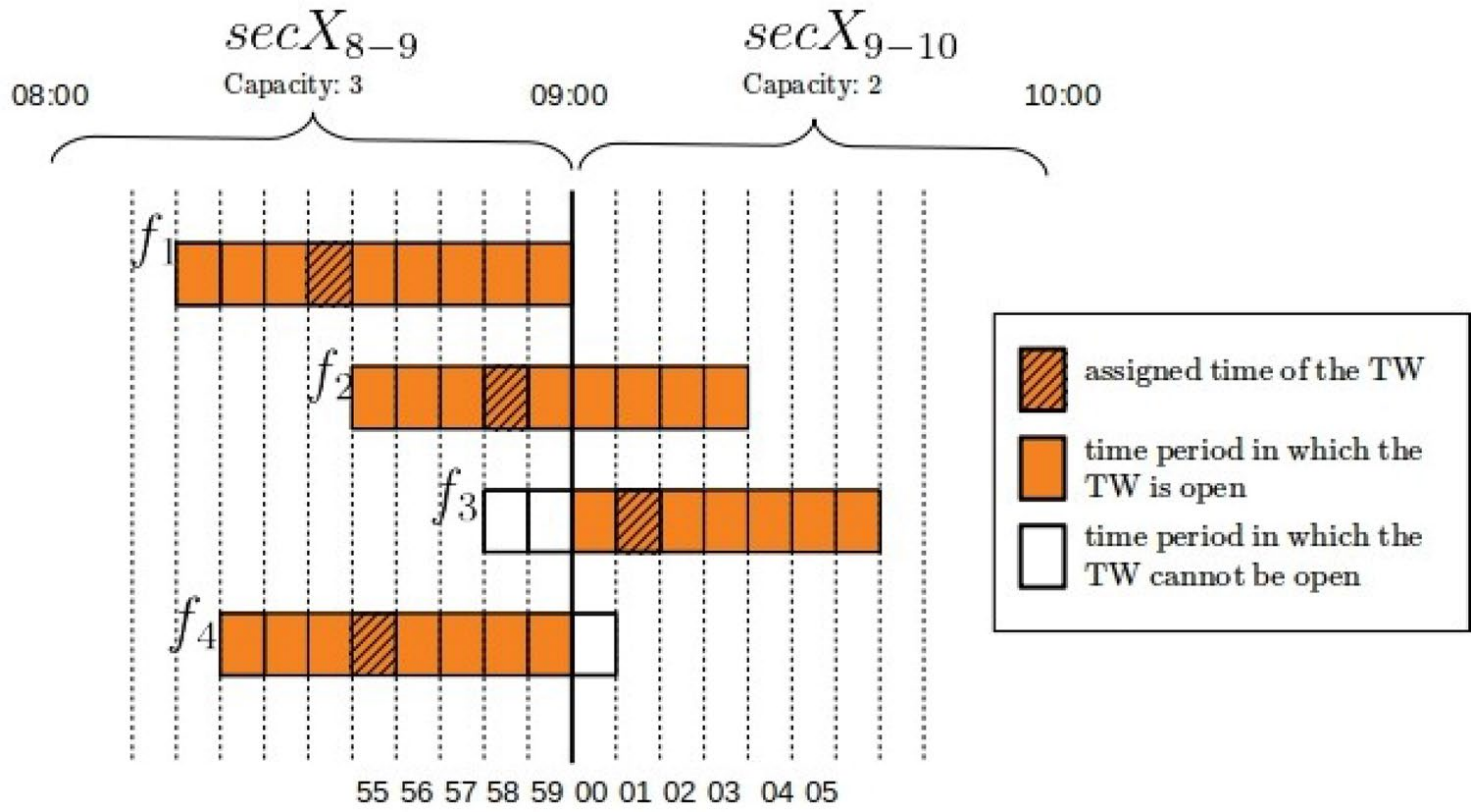
Depiction of the TWs of four different flights entering the same sector on the threshold between two sectors-hours

The second, TW model, evaluates and assigns a measure of flexibility to each flight trajectory. The flight flexibility is expressed in terms of time windows, which are defined as time intervals associated with each flight operation (departure, arrival or entry into sectors along the route). A TW is characterised by the assigned time and duration (see Fig. 1 in the following subsection for illustration). The departure time, sector entries and arrival time as computed by the SATA model become the input in the TW model and represent the assigned times of the TWs along the trajectory. Given all TW assigned times, the maximization of the TW duration is carried out by means of an integer linear programming problem, which extends the model introduced in [23] for the tactical setting. Notation, mathematical formulation and a detailed description of the decision variables, objective function and constraints is given in [22], which also explores different variants of the TW model. In this work, we discuss only one model variant—conservative TW model with asymmetric TW extension, where the TWs are extended backward and forward from the assigned time, with the different duration of backward and forward extensions: $-\,5$ and $+\,10$ min, for total duration of 15 min.^6^

As long as the flight operation is performed within the duration of assigned time window, the flight will not cause disturbances to other flights in the system (e.g. delay). The goal of the TW model is to maximise the overall flexibility among all the flights, subject to the capacity constraints of the network elements. In case a flight traverses a highly congested airspace, where significant inter-dependencies between flights exist, even a ‘small delay’ could cause a large downstream effect. These flights are referred to as constrained. Conversely, a flight is deemed unconstrained when it traverses a non-congested area, where its delay would have impact only on the flight itself. With this in mind, the duration of a time window is a measure of the flexibility that can be granted to the flight: the longer the time window, the greater the flexibility. Unconstrained flights can be given ‘large’ time windows, while the constrained flights have less flexibility and are characterised by ‘narrow’ time windows.

Outputs of the TW model allow the identification of constrained and unconstrained flights, as well as the distribution of congestion in space and time throughout the European network, at the strategic / pre-tactical phase. This could be an important input into future strategic collaborative flight/traffic planning, as the hotspots and flight flexibility are identified in advance. Airlines can obtain the information on the problems in the network and the ANSPs and the Network Manager could try to adjust the ATM system performance through the implementation of mitigation actions.

### Time windows

In this section we illustrate the concept of TWs. Figure 1 depicts the TWs of four different flights for which entry into the same sector *SecX* is expected around 10:00^7^; for these flights the assigned time of entry (TW assigned time) is represented by the shaded rectangles. In this example, each of the entries could be performed up to 3 min before, or up to 5 min later than the assigned time. Therefore, the maximum duration of the TWs equals 9 min. The orange rectangles show the time periods in which the TW is open, where the associated sector entry can be performed; the white rectangles show the time periods in which the TW cannot be open due to the capacity constraints of the sector. The capacity is defined as the number of entries into the sector during that hour. Each network element can have different capacity, and for simplicity sake we refer to the capacity of an element as sector-hour capacity (even for the airport capacities).

In the example in the Fig. 1, the sector *SecX* is active (i.e. open) from 9:00 to 11:00. During the sector-hour $SecX_{9-10}$, from 9:00 to 10:00 the capacity is 3, while in the following sector-hour $SecX_{10-11}$ a capacity is 2 entries per hour. The assigned times of the TWs of flights $f_1$, $f_2$ and $f_4$ for entry into sector *SecX* are in the sector-hour $SecX_{9-10}$ within which, more than 3 entry operations are not sustainable. For this reason, the TW of flight $f_3$, can extend backwards only up to 10:00, and cannot cross in the previous sector-hour, otherwise the capacity constraint would be violated. Since flights $f_1$, $f_2$ and $f_4$ have already reserved a unit of capacity to enter $SecX_{9-10}$, their TWs can be extended within the same sector-hour without limitation. The assigned time of flight $f_3$ is in the sector-hour $SecX_{10-11}$ within which 2 entries are allowed, thus another flight can be allowed entry from 10:00 to 11:00. Only $f_2$ or $f_4$ can be allowed to enter within sector-hour $SecX_{10-11}$, in which case the model favours the entry of $f_2$ because it leads to a solution with greater overall flexibility, that is to say the total number of time periods in which all the TWs are open.

Finally, note that for a given flight, the duration of the TWs may vary depending on the area in which the action is carried out. For example, the departure airport could be in an area that is not very congested, but very crowded portions of airspace must be crossed during the flight. Since the flexibility of a flight is obviously constrained by the minimum TW duration, we impose the same duration on all TWs of a flight, equal to the minimum one. Of course, different flights can have TWs of different duration.

### Data instance and computation times

The two models are run on a day of real air traffic data, encompassing the entire European Civil Aviation Conference (ECAC) airspace. Different data items are needed to run the models, including flights, airspace configuration, capacities of resources (sectors and airports), trajectories, aircraft types and their operational costs, fuel costs, route charges (unit rates), and airline types. The data on air traffic and air network structures are sourced from EUROCONTROL’s Demand Data Repository 2 (DDR2). Cost data are taken from the [24] report. As already mentioned, we use sector-hour capacity: hourly number of entries in the sector, when that particular sector is active or rather when the configuration it belongs to is active. Furthermore, the airport capacities are used, and they can be defined for arrival, departure or general (mix of arrival and departure) operations. The capacity is given as a number of such operations within an hour.

The data instance is created with the traffic from September 1st, 2017, a busy, but not unduly disrupted day in September 2017. This day is ranked as the fifth busiest day in 2017, but with significantly lower ATFM delay with respect to the days with higher traffic in 2017. The data instance creation is described in more detail in [22]. The instance takes into account 29, 917 flights and more than 24,000 sectors-hours (derived from 1458 sectors and 204 airports active on September 1st, 2017).

In our computational experiments, we first run the SATA model which assigns the trajectories and departure times to all flights. Then we run the TW model to determine the flexibility (i.e. TW duration) of each flight. Computational experiments were run on a 64 bit Intel(R) Xeon(R) E5520 @ 2.27GHz quad core CPU computer, having 16GB of RAM memory and Debian 8.0 operating system. The optimality gap for TW model was set to 0% and maximum TW width was set to 15 (up to 5 min before and 10 after the assigned time). The computation time for SATA is around 300 s, while it is 5 s for the TW model.

## Data visualization

**Fig. 2:**
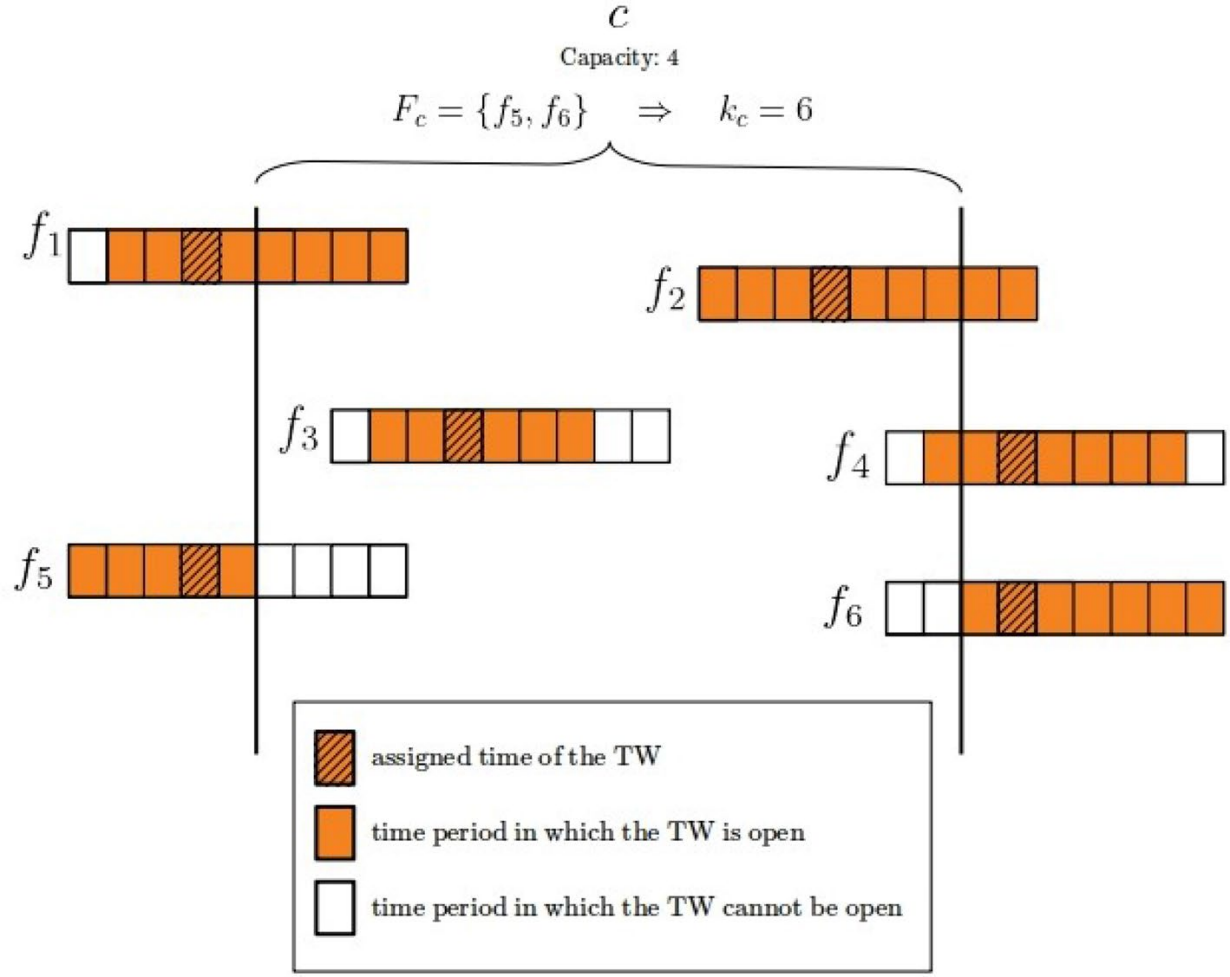
Example of the criticality index calculation

In this section we describe the visualization tool developed to share the results of the above described models in the form understandable to different stakeholders. Before describing the tool, we need to introduce a few definitions:

- *Constrained flight* is a flight *f* that is assigned a TW of duration $w_{f}$, which is shorter than the maximum TW $w_\mathrm{{max}}$.
- *Saturated or critical sector-hour*^8^—a sector-hour where some (constrained) flights cannot reserve capacity for earlier or later execution of their corresponding operation because the capacity limit has been reached.
- *Criticality index* $k_\mathrm{c}$ measures the degree of criticality of a sector-hour as the total additional number of the time periods that all flights constrained by the same sector-hour would have if it had sufficient capacity. On the whole, the criticality index of a sector-hour is overestimated as the constrained flight could be limited by multiple sector-hours along its trajectory. The criticality index $k_\mathrm{c}$ is:

$$\begin{aligned} k_\mathrm{c}=\sum _{f \in F_\mathrm{c}} (w_\mathrm{{max}}-w_{f}), \end{aligned}$$

where $F_\mathrm{c}$ is the set of constrained flights that have TW duration constrained by the used-up capacity of the saturated sector-hour c. In the example in the Fig. 2, 6 flights may reserve a unit of capacity in the constrained sector-hour *c* during which the capacity is 4 entries per hour. $f_1$, $f_3$, $f_4$, $f_5$ and $f_6$ are constrained flights since they have been assigned a TW of duration shorter than the maximum. However only $f_5$ and $f_6$ ($\in F_\mathrm{c}$) cannot reserve a unit of capacity in *c* while $f_1$, $f_3$ and $f_4$ are constrained from other saturated sector-hours. Therefore the criticality index $k_\mathrm{c}$ adds up 4 and 2 time periods during which *c* is open and the TW of $f_5$ and $f_6$ respectively cannot be open.

**Fig. 3:**
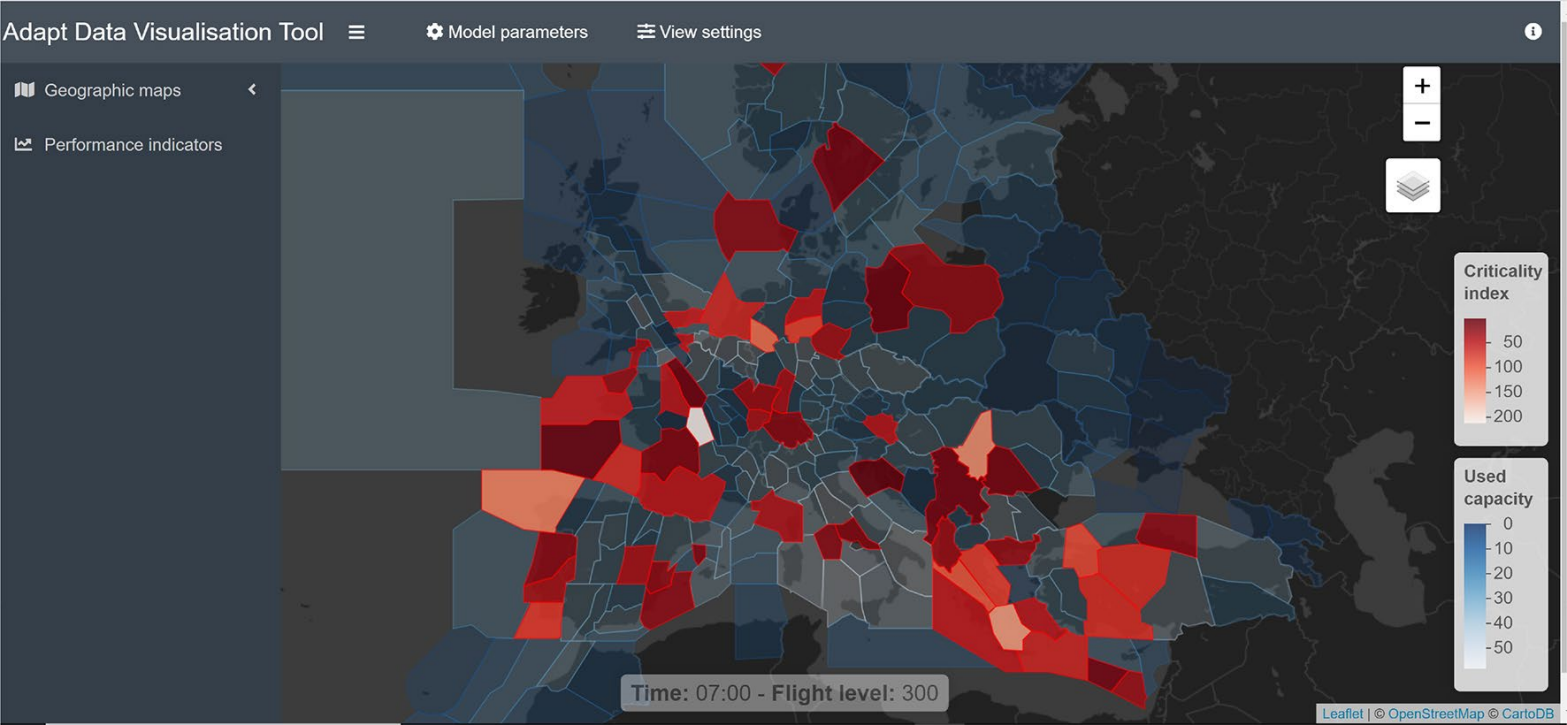
The visualization tool

The data visualization tool can be found at https://visualization.adapt-h2020.eu/. Different pieces of information can be obtained from the tool, both on flights and on airspace/airports. Let us start with the short description of different options of visualization. Figure 3 shows the main screen. At the top, there are two “buttons”: model parameters and view settings. The model parameters allows to choose the visualization of the results of different variants of TW model^9^. The View settings button opens a menu for flight level and time of day choice, as well as the capacity depiction. On the left hand side we have Geographic maps and Performance indicators “buttons”. Geographic maps offers the view by Sectors (default), ACC/FIR, ACC^10^ hourly maps, Bridging and detaching sectors, and B&D sectors hourly maps. At the main screen, the map of sectors is shown, the time of day and flight level given at the bottom of the screen. In the bottom right angle, we have the legends.

**Fig. 4:**
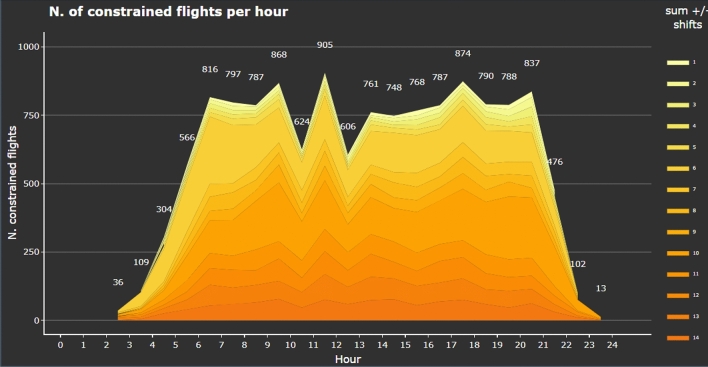
Distribution of constrained flights across the day

Let us turn to exploring the information coming from the modelling output. Let us start with the information on constrained flights. With the TWs of 15 min (5 min before the assigned time to 10 min after), the TW model identifies 13, 362 constrained flights, representing the 44.7% of the total number of flights. Figure 4 shows the number of constrained flights per hour and TW duration (the darker the colour the larger the TW). It can be seen that the number of extremely constrained flights (i.e. TW of 1 min) is very low ($< 1 \%$) and that the largest number of constrained flights have TWs of 10 min (9.4%), followed by TWs of 6 min (8.5%). Most of the flights that have a TW of 6 min can operate their flight 5 min earlier or at the assigned time. The peak at 10 min covers mostly the flights that cannot start earlier, but could delay up to 10 min after the assigned time. The graph can be found under the Performance indicators.

**Fig. 5:**
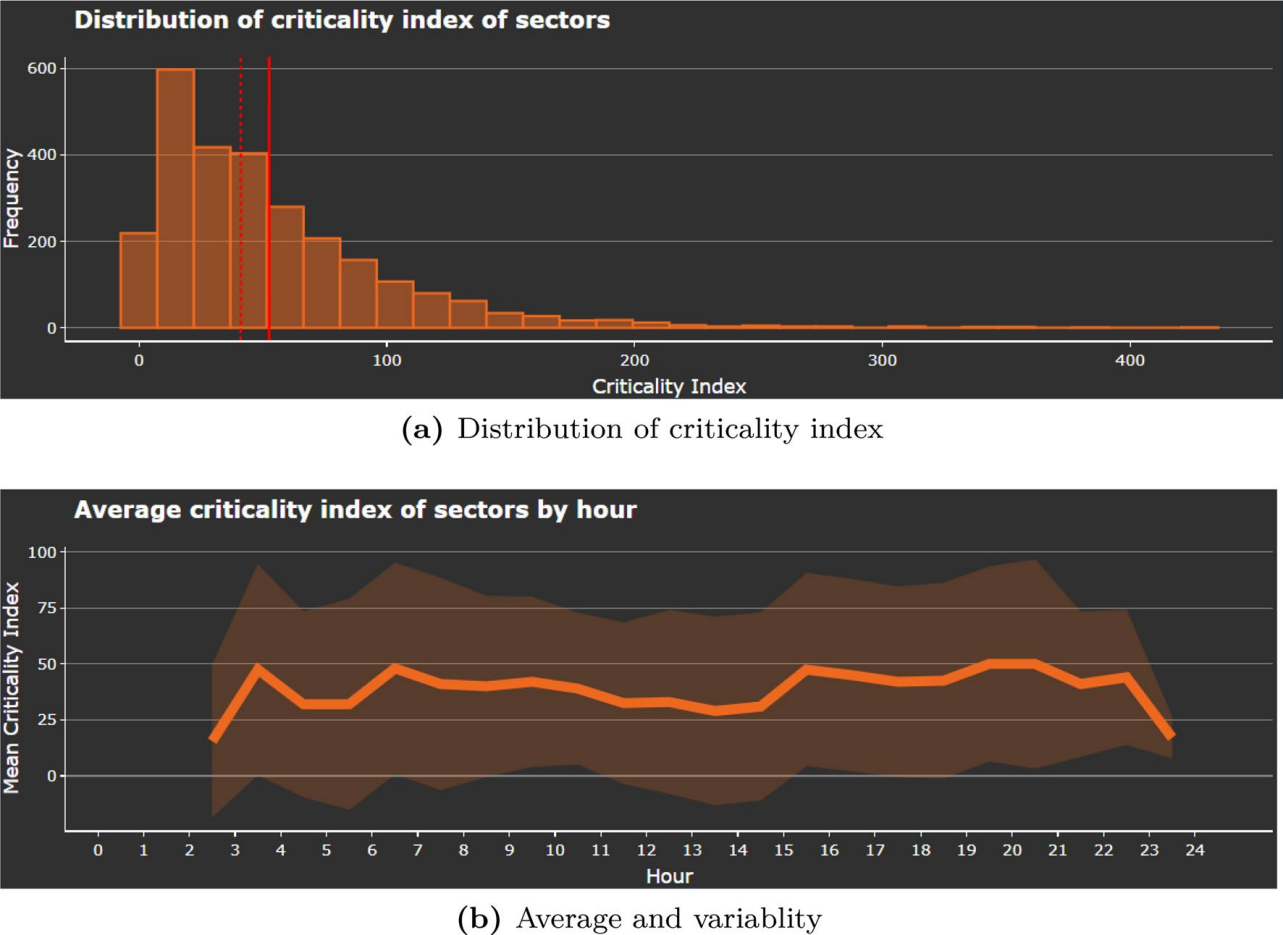
Distribution of criticality index across sector-hours and average and variability of the criticality index across the hours of the day

**Fig. 6:**
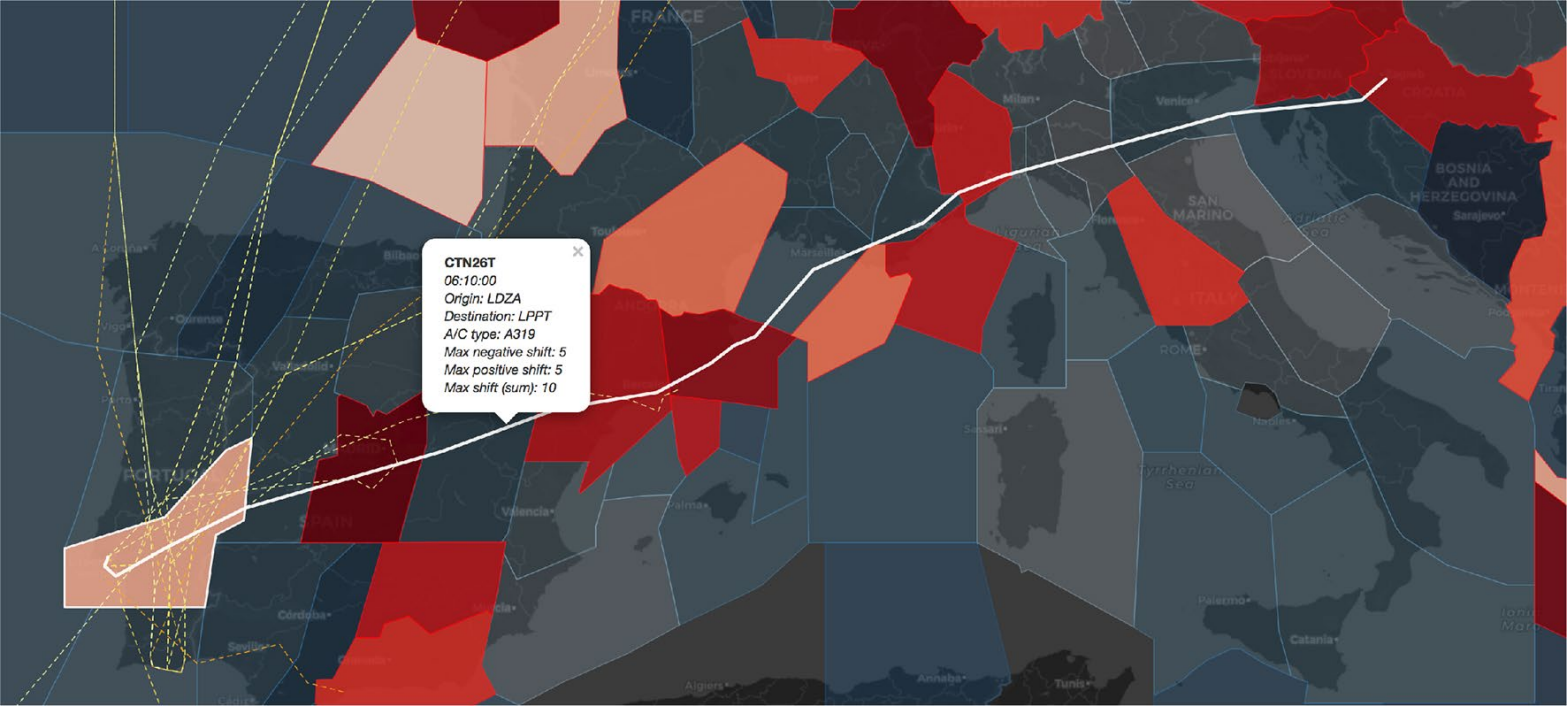
Flight from LDZA (Zagreb) to LPPT (Lisbon), constrained by 6 sector-hours

Figure 5a, b represent two further graphs that can be found under Performance indicators. An analysis of the criticality index of the saturated sector-hours shows that the vast majority of sector-hours exhibit low values, albeit with a few notable outliers (Fig. 5a), with the average and standard deviation being fairly constant throughout the day (Fig. 5b).

Apart from the overall flight flexibility, we can identify the network elements that impose limits on particular flight’s flexibility. Figure 6 shows a constrained flight trajectory that crosses six saturated sectors (i.e. sector-hours) that constrain this flight to a TW of 10 min, whereas the dashed lines represent the trajectories of other constrained flights (i.e., TW duration lower than 15 min) that cross sector LPPCCEL from 09:00 to 10:00.

**Fig. 7:**
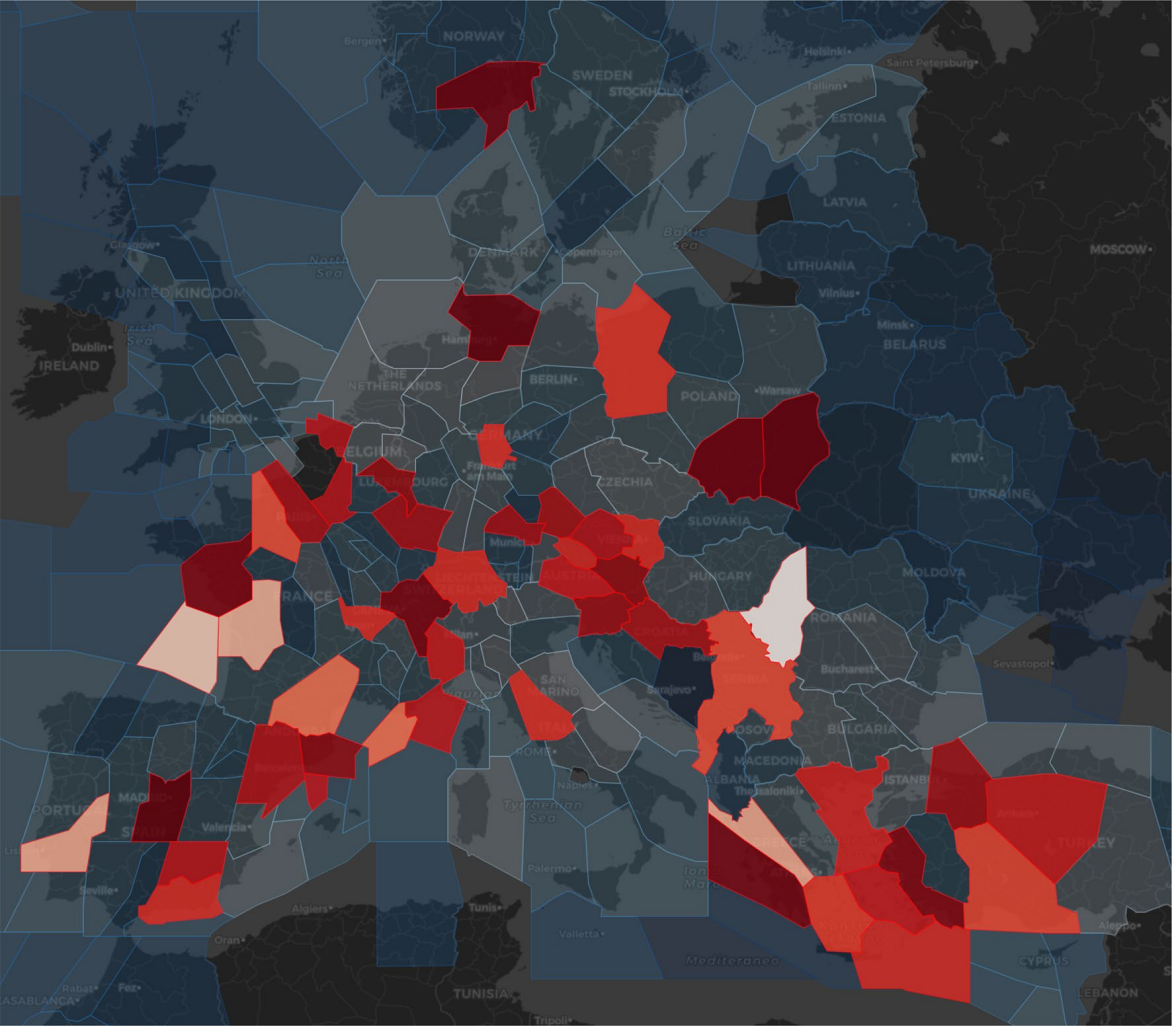
Critical sectors between 9:00 and 10:00 at FL300

**Fig. 8:**
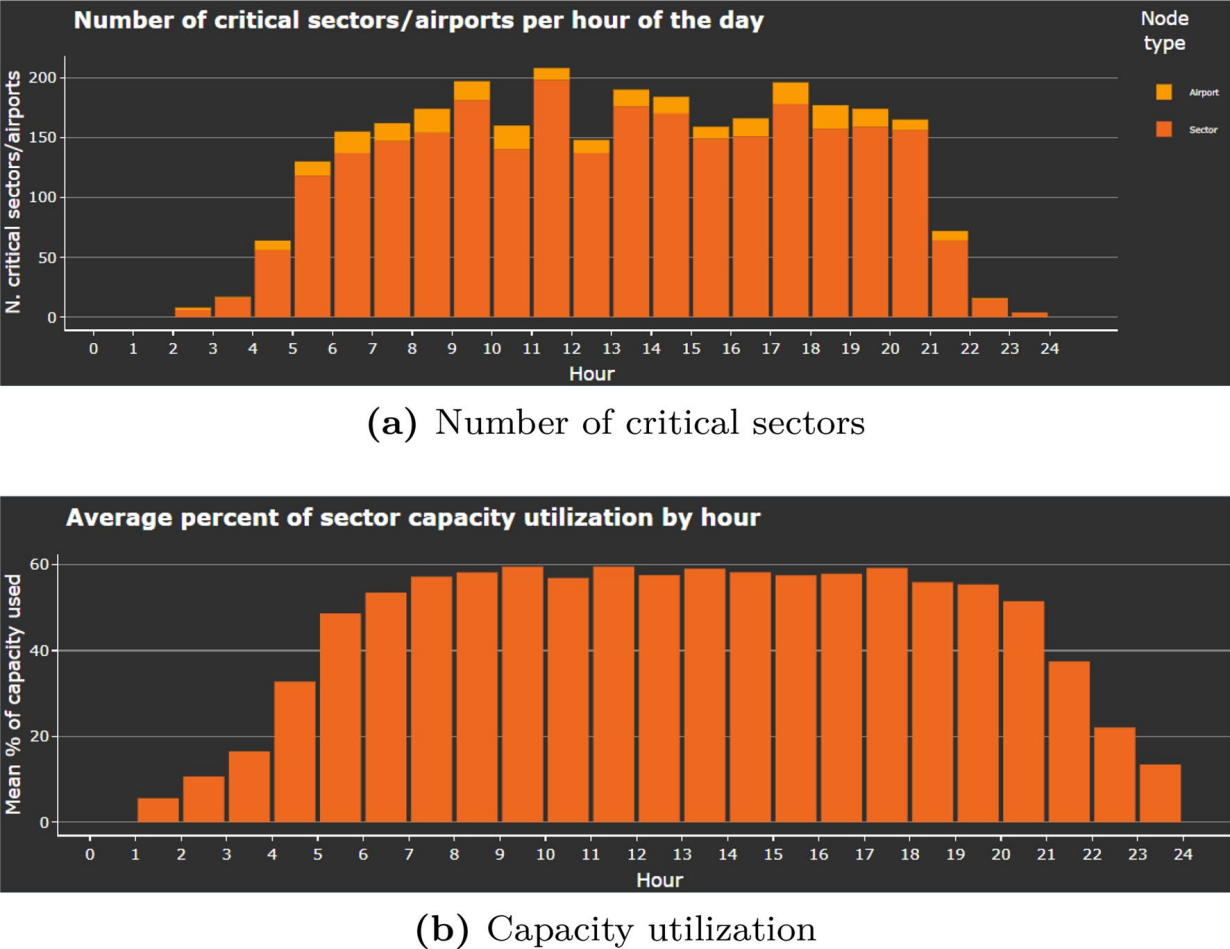
Graphs showing information on the airspace under analysis- number of critical sectors (top) and average capacity utilization (bottom)

Let us now turn to the information on the capacity usage in the network. Figure 7 depicts an example of geographical location of saturated sectors at flight level 300 in the hour between 09:00 and 10:00 (the lighter the colour, the higher the criticality index). Figure 8a shows the number of critical sectors/airports by hour, and the 8b shows average percent of capacity utilization per hour. Overall, 2926 sector-hours were saturated during the day, out of approximately 24,000 sector-hours open during the day.

Relatively low number of saturated sector-hours, average percent of sector capacity utilization that is lower than 60%, low percentage of very constrained flights, and the distribution of critical sector-hours lead us to the conclusion that even on a very busy day like our test day, the difficulties on the network that lead to a limitation of flight flexibility are distributed both spatially and temporally. Thus, an improvement in the performance of the entire system could be achieved with specific interventions, as will be shown in the following section.

## Example of use through ANSPs’ mitigation actions

The actual capacity of an ANSP^11^, and consequently its Area Control Centres (ACCs), at each point in time depends on the applied configuration/s. A configuration consists of a number of sectors. The higher number of sectors in a configuration is usually indicative of higher capacity of the airspace applying the configuration. The supervisor choses a configuration, depending on the traffic demand prediction (today, the prediction is the short-term one, based on the submitted flight plans) and the staff availability. The configuration changes when the demand requires it, at any time of day. ACCs usually change the configurations a number of times throughout the day, to best match the changing traffic demand. The information on the saturated sectors and their criticality indices can be used by ANSPs to formulate mitigation actions to improve the situation [22]. For example, an ACC with a small number (i.e. one or two) of saturated sectors, having the low criticality index, might decide to keep the current configuration. Even if the capacity is breached, it might be for a small number of flights, which often happens in every-day operations. Nevertheless, if several sector-hours within an ACC have high criticality indices, the configuration change to the higher capacity one might be the decision to take. Thus, the ANSP mitigation actions we focus here regard the increase of ACC capacity through the change of configuration.

**Fig. 9:**
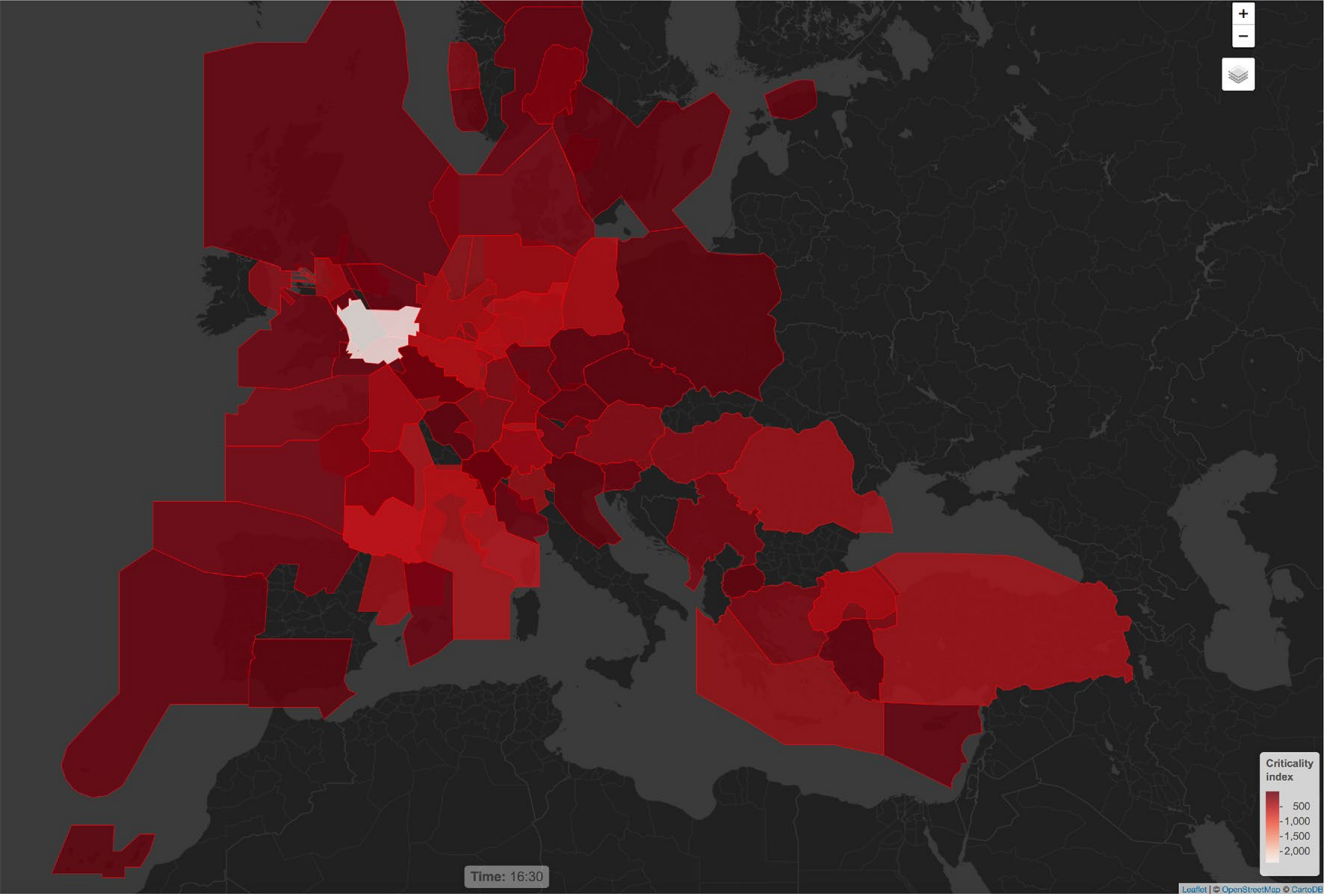
ACC-hour criticality index from 16:00 to 17:00

In order to ease the decision-making process (see [22]), the aggregate criticality index $k_\mathrm{{a,h}}$ is introduced. The index contains the degree of criticality of all saturated sector-hours relating to an ACC a in the hour h (we define this as ACC-hour). The aggregate criticality index is a simple sum of the criticality indices $k_\mathrm{c}$ grouped by ACC-hour:

$$\begin{aligned} k_\mathrm{{a,h}}= \sum _{c \in SH_\mathrm{{a,h}}} k_\mathrm{c}, \end{aligned}$$

where $SH_\mathrm{{a,h}}$ is the set of saturated sector-hours within ACC a during the hour h. The high aggregate criticality index identifies a time interval within which the chosen ACC configuration does not guarantee appropriate level of capacity to meet the traffic demand. For instance, Fig. 9 shows the ACC-hour criticality index from 16:00 to 17:00 of the entire ECAC area (the lighter the colour the higher the criticality index).

We want to showcase the possible use of the strategic traffic planning and flight flexibility concept and the visualization tool through the assessment of mitigation actions by a few ANSPs increasing the capacity of their saturated airspace. Here, we show the impact of our choices (described in detail below), but we do believe that this type of action should be left to experts (i.e. supervisor) that are more familiar with their airspace and what is more important, with staff availability. The TW model results (i.e. saturated sector-hours, criticality indices) can help experts in their decision-making process. Their choices can then be fed into the model again, and test its impact on a single ACC, and the network.

In general, the mitigation action through the change of airspace capacity should observe the following steps. First, the hourly criticality indices of ACCs are ranked from the highest to lowest, accompanied by the configuration in use at that hour. In the next step, the availability of higher capacity configurations is checked. Keep in mind that the availability depends on the technical (i.e. the ACC has a higher capacity configuration) and human resources (i.e. there are enough air traffic controllers to open all the sectors in the higher capacity configuration at that hour). Further, a choice is made for a higher capacity configuration in certain ACC-hours, which is then fed into SATA and TW models to assess the impact on those ACCs and the network as a whole.

In order to propose a mitigation action in our example, we first identify the most critical ACC-hours and then assign an alternative configuration, if a configuration with higher capacity exists in that portion of airspace. Then we run SATA and TW models again; SATA model gives trajectories and initial TW times, TW model gives the flexibility measure (TW duration), with the new capacity values. Table 1 shows the ACC-hours with highest aggregate criticality index: for each ACC-hour the number of saturated sector-hours, constrained flights, and the criticality index is shown.

**Table 1:** ACC-hours with highest aggregate criticality index

ACC	Hour	Saturated sh	Constrained flights	Criticality index
LFBBCTAS	09:00–10:00	11	147	990
LSAGUTA	19:00–20:00	11	113	861
LECPCTA	07:00–08:00	10	116	831
EDWWCTAE	05:00–06:00	8	93	758
EDWWCTAE	19:00–20:00	8	103	736
EDYYBUTA	13:00–14:00	8	103	684
LFBBCTAS	19:00–20:00	8	107	677
LEBLTMA	07:00–08:00	6	91	659
LFBBCTAS	07:00–08:00	9	96	624
EDYYBUTA	14:00–15:00	4	82	607
EDYYBUTA	19:00–20:00	8	92	598
LFBBCTAS	17:00–18:00	5	73	564
EHAACTA	07:00–08:00	5	80	549

**Fig. 10:**
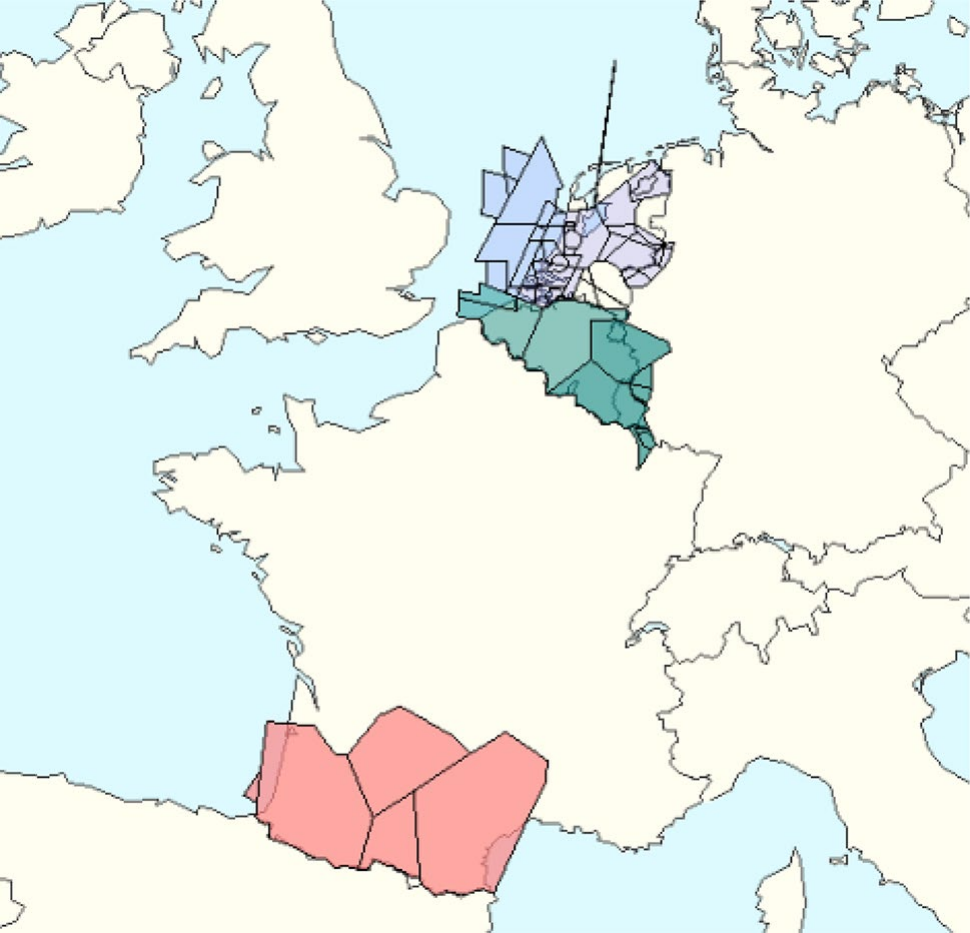
Analysed ACCs: LFBBCTAS (red), EDYYBUTA (green), EHAACTA (blue), source (EUROCONTROL’s NEST Tool)

**Fig. 11:**
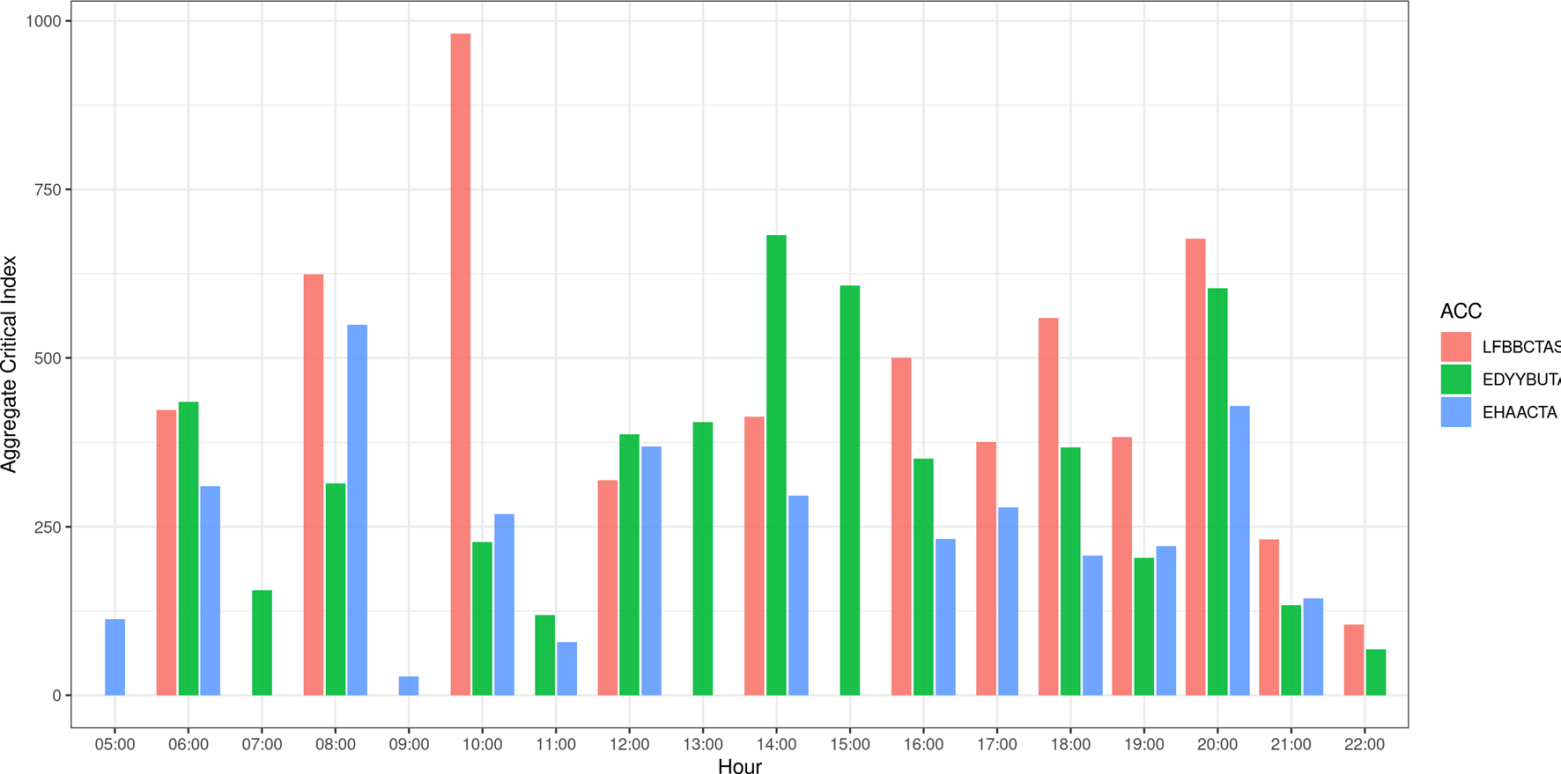
Value of the aggregate criticality index for each of the three analysed ACCs, during the hours of the day

For mitigation action we focus on: LFBBCTAS, EDYYBUTA and EHAACTA ACCs. The aggregated criticality indices of these ACCs are among the highest among ECAC ACCs. Furthermore, their central geographical position, and the amount of traffic they control, identifies them as significant portions of European air traffic network. Figure 10 shows the geographical position of the three chosen ACCs, while Fig. 11 shows the changes in the aggregate criticality indices during the hours of the day.

To illustrate the process of choosing the alternative configuration, we will take the ACC EHAACTA as an example. Table 2 shows the configurations that were in place during the test day, with the related opening hours. We focus only on the configurations that were in use on that day, assuming that the availability of the controllers was a factor in their choice, and thus do not take into account other configurations even if they would possibly offer higher capacity.

**Table 2:** Configuration and opening schemes chosen for EHAACTA

Configuration	Number of sectors	Opening time
CONF1	2	00:00–04:19
CONF5	3	04:20–04:59
CONF3	5	05:00–05:19
CONF4	6	05:20–17:39
CONF3	5	17:40–17:59
CONF2	4	18:00–20:39
CONF5	3	20:40–20:59
CONF1	2	21:00–23:59

From the table can be seen that the configuration CONF4 has the most sectors (on that day), which therefore offers greater capacity, and is active from 5:20 to 17:39. This period covers the peak in the aggregate criticality index (549) that occurs around 8:00. In this time interval, therefore, the assignment of a different configuration would entail lowering of the capacity and for this reason a worsening of the performance in terms of aggregate criticality index. The Fig. 11 shows that around 20:00 the ACC has high aggregate criticality index, while from the Table 2 can be seen that the configurations open in that period have lower capacity that the CONF4. Therefore, we chose to open the configuration CONF4 also in the period from 20:00 to 22:00.

**Fig. 12:**
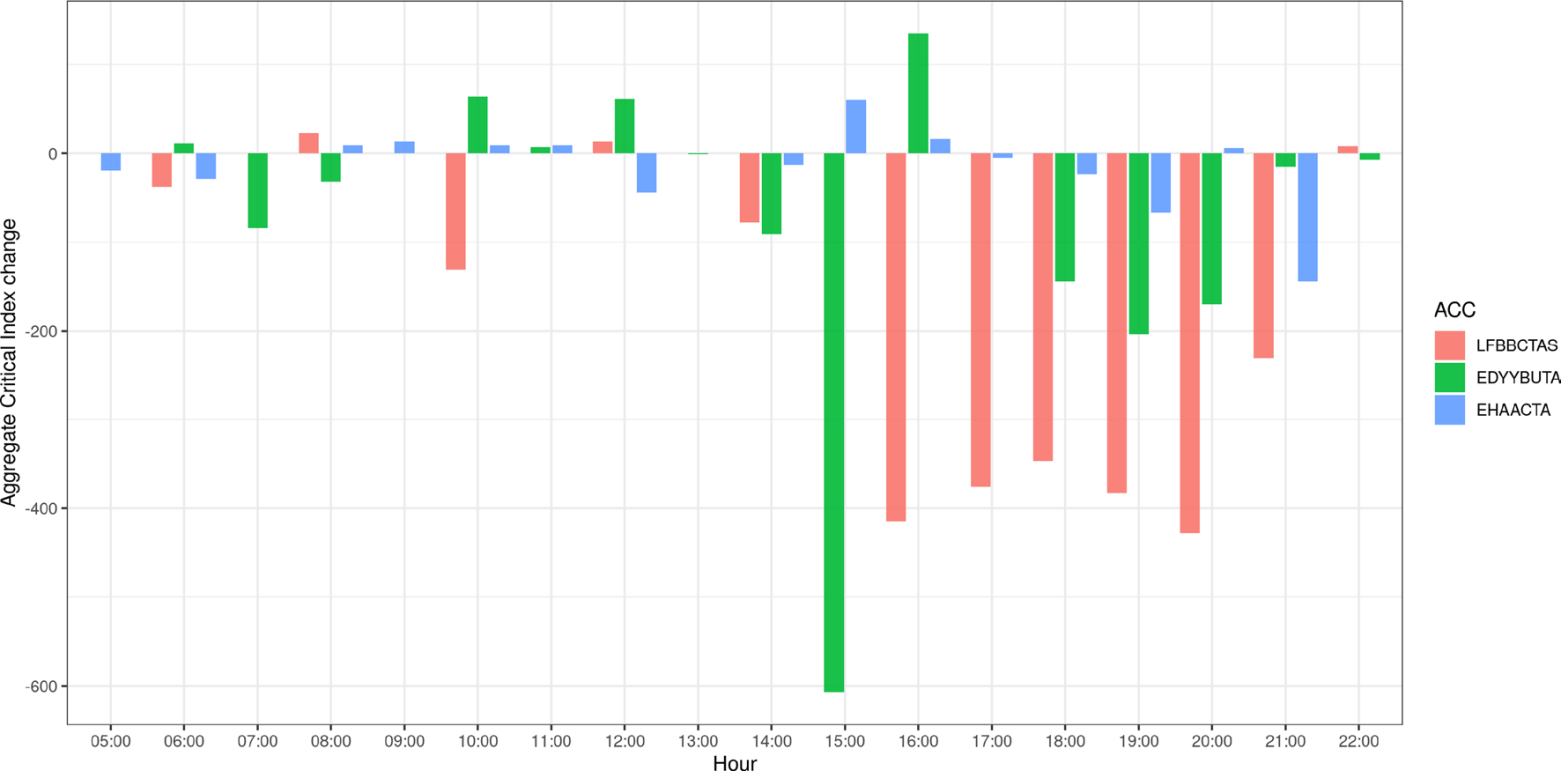
The change in the value of the aggregate criticality index for each of the three analysed ACCs, during the hours of the day

Following the same reasoning we proposed modifications of the existing configurations for critical time periods for the other two ACCs. The configuration proposed as an alternative is always the one that offers the maximum capacity among those assigned on the same day. For LFBBCTAS we chose the configuration 9S.2, which consists of 9 sectors, in time slots 10:00–11:00 and 16:00–21:00. For EDYYBUTA we chose the configuration 6.2, which consists of 6 sectors, in time slots 06:30–07:00, 15:00–15:30 and 18:00–19:30.

**Fig. 13:**
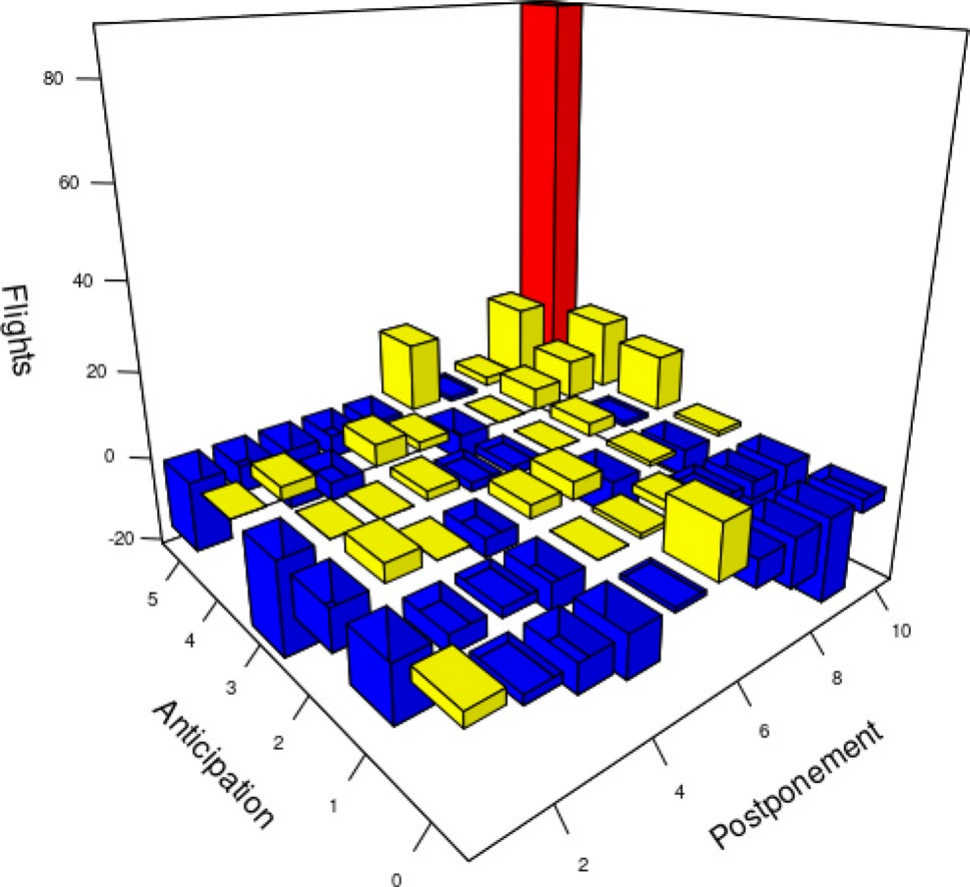
The change in the number of constrained flights across the assigned TW durations, after the configuration change

Figure 12 shows the change of the aggregate criticality index for the three chosen ACCs, across the hours of the day. As expected, in the ACCs in which we changed the configuration for the higher capacity one, there was a significant decrease in the value of the aggregate criticality index. However, a more interesting result to analyse is the effect the mitigation action has on the air traffic in the network, shown in Fig. 13. The axis labelled “Anticipation” shows the TW duration before the assigned time, while the “Postponement” axis shows the TW minutes after the assigned time. The vertical, flights axis depicts the change in the number of flights assigned to each of the TW value categories after the configuration change. There is a significant increase in the number of non-constrained flights, which is depicted by the red column at the far end of the graph ($-\,5$, 10 min category, denoting non constrained flights). This increase is due to the decrease in the number of flights characterised by a shorter duration of the time window, depicted by blue columns. The yellow columns show the increase in number of flights in that category. We can see that overall, the flights gained in TW duration as the blue columns that present decrease of number of flights with respect to the initial solution are mostly found in the shorter TW categories, while the yellow columns that gained flights have longer TWs (see for example the columns $-\,$5 to 9, $-\,$4 to 10). As can be seen from these two indicators, even a conservative configuration change can increase the capacity to such levels to decrease the criticality of saturated sector-hours and at the same time increase the flexibility of flights.

## Assessment of mitigation action results

In order to further evaluate the solutions obtained from the models and the mitigation action, a baseline scenario is defined for comparison. A suitable baseline scenario is obtained by applying the strategic model (SATA), with unconstrained capacities, which is consistent with the current practice of not considering capacity in the strategic phase. Baseline scenario de facto corresponds to a simple assignment of routes of minimum cost, disregarding capacities. Thus, the baseline scenario assigns minimum cost routes (from a set of possible routes) to flights, at the requested departure times. Some arrival shift is possible (if the chosen route is longer than the shortest duration route). As the capacities are not enforced, the TW model cannot be applied (as in this case the TWs would be either infinitely large, or of the maximum allowed duration). Thus, the measure of flexibility cannot be obtained in the baseline scenario, which is also consistent with the current situation. Solution scenario consists of the application of the SATA and TW models on the created data instance (see Sect. 2.2). Mitigation scenario consists of the application of the SATA and TW models on the data instance to which the configuration changes proposed in the Sect. 4 have been made.

The following indicators are taken into account in this assessment:

- Departure shift: absolute difference between the requested and assigned departure time.
- Arrival shift: absolute Difference between the arrival time obtained by departing at requested arrival time using the shortest route and the assigned arrival time.
- Flight strategic operational costs: cost of flights operations calculated considering the assigned routes (airborne, fuel) and strategic shifts (ground costs).
- Route charges per flight: route charges imposed on the flights, used to cover the costs of Air Navigation Service (ANS) provision.
- Horizontal en-route flight efficiency: ratio of great circle origin-destination distance over the en-route distance between the origin and destination.
- Temporal flight efficiency: ratio of the duration of the shortest route over the duration of the assigned route.
- ANSP revenues: sum of all the charges each flight needs to pay to pass through the ANSP’s airspace.
- Sector capacity utilization. This indicator shows for each open sector the capacity utilization, measured as the number of sector entries over the declared capacity during the chosen time interval (e.g., 1 h).

On these indicators we can compare the flight trajectories generated by the SATA model before and after the proposed mitigation action, as well as the comparison with the baseline scenario. Table 3 lists the value of indicators for the baseline, solution and mitigation scenarios and relative percentage differences. Note that the capacities are not respected in the baseline scenario, while the solution and mitigation scenarios redistribute flights, so that the capacities are respected.

**Table 3:** Value of assessment indicators across the three scenarios and relative percentage differences

	Baseline	Solution	Mitigation	Sol-base (%)	Mit-Sol (%)
Departure shift (min/flight)	0	1.42	1.36	–	$-$ 4.22
Arrival shift (min/flight)	0.14	1.80	1.74	1200	$-$ 3.33
Shifted flights	0	4931	4771	–	-3.24
Airborne costs (e/flight)	2942	2949	2950	0.24	0.03
Fuel costs (e/flight)	2871	2878	2879	0.24	0.04
Ground costs (e/flight)	0	13	13	–	0.00
Route charges (e/flight)	728	731	731	0.41	0.00
Horizontal efficiency	0.9338	0.9329	0.9328	$-$ 0.10	$-$ 0.01
Temporal efficiency	0.9987	0.9957	0.9955	$-$ 0.30	$-$ 0.02
ANSP revenues (millions)	21.22	21.28	21.28	0.28	0.00

For the capacities to be respected the solution scenario needed to shift 4931 flights (16% of the total of 29, 917 flights in the data instance), and the arrival and departure shifts are lower than 2 min per flight. The costs in general increase, but on the order of a few euros per flight. The mitigation scenario resulted in lower number of flights needed to be shifted, and lower departure and arrival shifts, while the flight costs increased very little with respect to the solution scenario. This is also to be expected as the higher capacity in general offers more breathing room for all the stakeholders. For both solution and mitigation scenarios the new trajectory distribution is a little bit less efficient both horizontally and temporally compared to the baseline scenario.^12^ As the route charges increase, the ANSP revenues increase about 0.2%. Here we need to consider that this is one of the busiest days in the year, and that the baseline trajectories most likely passed through ANSPs with lower unit rates.

**Fig. 14:**
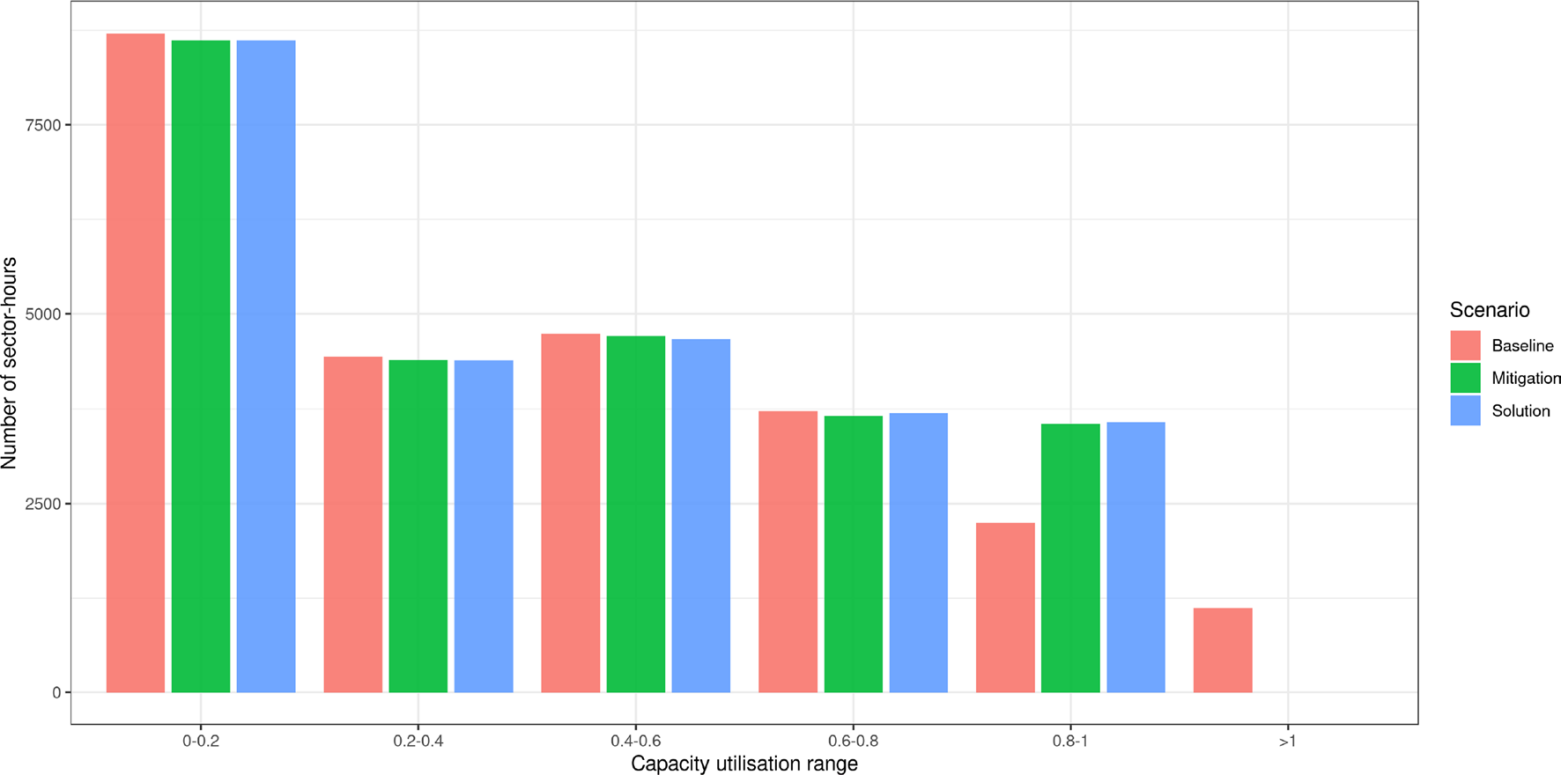
Distribution of sector capacity utilization across scenarios

Figure 14 shows the distribution of sector capacity utilization across all scenarios. We can see that for the great majority of sector-hours the capacity utilization is low ($<60$%). There is relatively small number of sector-hours that are close to saturation and saturated ($\ge 100$%). In the baseline, about 1000 sector-hours have capacity utilization higher than 100%. In all other scenarios there are no sector-hours where the capacity is breached, as that is imposed by the capacity constraints. Consequently, there are more sector-hours in the 80–100% category in the solution and mitigation scenarios with respect to those of the baseline—showing that traffic indeed was moved from congested sectors into those less congested. Note that the criticality index of the two adjacent ACCs under mitigation action was rather high, indicating they had little spare capacity. Thus, the redistribution of flights happened, but the closest sector-hours did not have too much spare capacity, so we have the noticeable impact only in the category of 80–100% capacity utilisation. The mitigation scenario resulted in slightly better capacity utilization, and slightly higher flexibility offered to flights (when compared to the simple solution scenario).

## Conclusions

This study allows to showcase a methodology for strategic traffic planning and a quantitative determination of the flexibility that can be attributed to a flight. We measure such flexibility in terms of time windows which are time intervals associated with each operation a flight must perform. If a flight is able to respect all the TWs assigned to it, all operations of any other flight are not affected. It is important to note that the measure of this flexibility is determined in the strategic phase of planning so as to allow different actors (ANSPs, airlines, airports, Network Manager) to take the appropriate countermeasures in time, if it appears that on the day of the operations there is a possibility that a certain sector/airspace becomes saturated, or a certain flight enjoys a very limited freedom of action.

Here we show how the results of such complex models can be shared with the stakeholders through the use of visualization tool, and how the tool can help in the decision-making process. Applying the SATA and TW models on real air traffic data for a test day (September 1st 2017) on the entire ECAC area, TWs were calculated for almost 30, 000 flights. We have seen that more than 50% of flights can benefit from TWs of maximum duration (15 min in this example) and that the percentage of very restricted flights (TW of less than 5 min) is very low ($< 5\%$). This means that at least on the day under investigation, European airspace could guarantee (from a nominal capacity point of view) ample room for flexibility in relation to the expected traffic demand for that day. In fact, we have seen that only 12% of the sector-hours constrains at least one flight, meaning that for the very large majority of sector-hours there are no nominal capacity issues in the strategic planning phase. However, if criticalities emerge during the strategic planning phase (i.e., the nominal capacity is not able to guarantee maximum flexibility to one or more flights), our models allow quantitative assessment of mitigation actions both in terms of increased flexibility of flights and criticality index reduction. In fact, we have seen that significant improvements have been made with just a few specific interventions, slightly modifying the airspace configuration of three chosen ACCs.

Model output visualization can increase understanding of the results by different stakeholders. Different views and performance indicators can further help stakeholders in formulating their decisions, when action is needed. Here, the focus was on the actions by ASNPs, but the same reasoning can be applied for the airlines and their flights. Moreover, it could be extended to other mitigation actions like the modulation of charges (i.e. demand-based pricing mechanisms).

## Data Availability

Data used is described in the Data Instance and Computation Times section. The results are available for visualisation at https://visualization.adapt-h2020.eu/.

## Footnotes

1 Source: https://www.eurocontrol.int/Economics/DailyTrafficVariation-States.html.

2 ATC capacity imposes constraints when foreseen traffic demand exceeds the declared capacity, while ATC staffing is applied when unplanned staff shortage reduces the planned capacity.

3 Strategic planning phase referred to in this paper extends from 6 months to several days before the operations

4 The model formulation is described in the paper http://dx.doi.org/10.1016/j.tre.2016.12.001.

5 Airspace configuration defines the (working) organisation of an area control center by specifying how the center’s sectors are combined to address the foreseen traffic (i.e. only one at low traffic demand).

6 Note that current, tactical ATFM slots are given in the same form. However, the meanings of the two are not the same.

7 Note that we use 24-h clock in the paper (00:00 format).

8 We use the terms saturated and critical sector-hours interchangeably.

9 Here we focus on conservative, asymmetric TW of 15 min, while the tool has also the intermediate and proportional variants, with forward, symmetric and asymmetric TWs.

10 Area Control Center.

11 Depending on the size, an ANSP can be composed of one or more ACCs.

12 Note that the trajectory choices used as input to SATA are coming from the last filed flight plans, not actually flown routes, thus the efficiency is lower than the actual efficiency numbers in Europe.
